# Caregiver-perceived behavioral challenges in fragile X syndrome and implications for measuring treatment benefit in clinical trials

**DOI:** 10.1186/s41687-026-01054-9

**Published:** 2026-04-01

**Authors:** Elizabeth Merikle, Nancy Tich, Terri Sebree, Randi Hagerman, Stephen O’Quinn, George Nomikos, Kristen Bzdek

**Affiliations:** 1 Harmony Biosciences, Plymouth Meeting, PA USA; 2 1323 Consulting, LLC, Johnson City, TX USA; 3 Medical Investigation of Neurodevelopmental Disorders (MIND) Institute, University of California Davis Medical Center, Sacramento, CA USA; 4 Department of Pediatrics, University of California Davis School of Medicine, Sacramento, CA USA; 5 Perissos Inc, Wake Forest, NC USA

**Keywords:** Aberrant behavior checklist, Cognitive interviews, Concept elicitation interviews, Content validity, FXS, Fragile X syndrome, Qualitative research

## Abstract

**Background:**

Fragile X Syndrome (FXS) is a rare X-linked genetic condition. Children with FXS exhibit a variety of challenging behaviors such as irritability, anxiety, and preference for solitary activities. The Aberrant Behavior Checklist-Community (ABC-C) is an observer-reported outcome (ObsRO) measure that captures many of the behavioral concerns (e.g., outbursts, social anxiety, difficulty connecting with others) salient to caregivers of individuals with FXS. An FXS-specific scoring algorithm of the ABC-C, considered more representative of the FXS phenotype (ABC-C_FXS_), has been included in numerous clinical studies of treatments for FXS. The goal of this qualitative research was to establish the content validity of the ABC-C_FXS_ domain structure in parents/caregivers of children with FXS and determine whether it is fit-for-purpose for measuring treatment benefit in clinical studies of FXS.

**Methods:**

The qualitative data came from 3 sources: (1) Concept elicitation (CE) interviews with caregivers of children with FXS (*N* = 10); (2) Caregiver survey (CS) (*N* = 212) completed at entry to CONNECT-FX (NCT03614663); and (3) Cognitive interviews of the ABC-C_FXS_ with caregivers of children with FXS (*N* = 25). Qualitative coding and analysis were consistent with best practices and followed principles in applied thematic (content) analysis. Item comprehension and relevancy of the ABC-C_FXS_ were also examined based on data from cognitive interviews.

**Results:**

The mean age of individuals with FXS was 10 (range 3–17) years and was consistent across the 3 data sources. The most frequently reported behavioral challenges described by caregivers were generally consistent across the CE interviews and the CS. Socially avoidant behaviors were most prevalent (70% and 49%, respectively) followed by socially unresponsive/lack of interaction (60% and 51%, respectively) and aggression (60% and 43%, respectively). Saturation of behavioral challenges related to FXS was reached in both the CE interviews and CS and was consistent with the content of the ABC-C_FXS_. Cognitive interviews showed that caregivers could understand and interpret the content and response options of the ABC-C_FXS_ and deemed its content relevant to their experience.

**Conclusions:**

The content validity evidence from this qualitative research supports the use of ABC-C_FXS_ as an efficacy measure in FXS clinical research studies.

**Clinical trial registration:**

Not applicable.

**Supplementary Information:**

The online version contains supplementary material available at 10.1186/s41687-026-01054-9.

## Background

Fragile X Syndrome (FXS) is a rare X-linked genetic condition with a prevalence of approximately 1 in 4000 males and 1 in 6000 females in the United States (US) [1]. FXS is the most common cause of inherited intellectual disability and the most common monogenetic cause of autism spectrum disorder. Children with FXS exhibit a variety of problem behaviors, such as anxiety, aggression, irritability, temper tantrums, hyperactivity, shyness, and preference for solitary activities [2, 3]. Proper measurement of the problem behaviors associated with FXS is needed to accurately demonstrate treatment benefit in clinical investigations of treatments for FXS [4].

The Aberrant Behavior Checklist-Community (ABC-C) is an observer-reported outcome (ObsRO) measure assessing inappropriate and maladaptive behavior in individuals with intellectual and development disabilities across 5 behavioral domains: Irritability, Social Withdrawal, Stereotypic Behavior, Hyperactivity/Noncompliance, and Inappropriate Speech [5, 6]. Previous research has examined the use of the ABC-C in individuals with FXS [7]. This work resulted in a modified domain structure and scoring of the ABC-C when assessing individuals with FXS, hereafter referred to as the ABC-C_FXS_. The most pertinent modification was the splitting of the Social Withdrawal domain into 2 domains: Social Avoidance and Socially Unresponsive/Lethargic. Given that socially avoidant behaviors are core aspects of the FXS behavioral phenotype [8], scoring of the ABC-C with a sixth domain (Social Avoidance) is considered more representative of the FXS phenotype than the original 5-domain scoring system of the ABC-C. Because the ABC-C captures many of the behavioral concerns (e.g., outbursts, social anxiety, difficulty connecting with others) salient to caregivers of individuals with FXS [9], it has been included in numerous clinical studies of potential treatments for FXS [10–13].

The patient-focused drug development initiative by the US Food and Drug Administration (FDA) emphasizes clinical trials should include data on treatment benefit from the patient’s perspective. Perspectives of those in close contact with the patient (e.g., caregivers) are commonly used to report the health status of children or other individuals who are not able to report reliably for themselves (e.g., individuals with intellectual disabilities such as found with FXS). Ideally, ObsRO measures such as the ABC-C include items that directly assess observed behavior [14]. The FDA systematically evaluates clinical trial endpoint measures, such as the ABC-C_FXS_, using established guidelines for determining whether the measure is fit-for-purpose^1^ [15, 16], which includes a review of the measure’s content validity within the intended patient population and context of use. Content validity is generally established using qualitative evidence demonstrating that a measure captures the most important concepts to patients and/or their caregivers, and that the clinical outcome assessment (COA) content (e.g., instructions and items) is relevant and well understood by respondents.

To date, no studies have examined the content validity of the ABC-C_FXS_ scoring algorithm in individuals with FXS. Accordingly, this qualitative research examines the content validity of the ABC-C_FXS_ domain structure in parents/caregivers of children with FXS to determine if the ABC-C_FXS_ adequately captures the behavioral problems associated with FXS and is fit-for-purpose for measuring treatment benefit in clinical studies of FXS.

## Methods

The qualitative data were gathered from 3 sources: (1) Concept elicitation interviews in primary caregivers of children with FXS (*N* = 10) conducted as part of a multi-phase research study [17]; (2) Caregiver survey (*N* = 212) completed at entry to CONNECT-FX (a randomized controlled trial of ZYN002 cannabidiol transdermal gel in children and adolescents with FXS (NCT03614663) [18]; and, (3) Cognitive interviews of the ABC-C_FXS_ in primary caregivers of children with FXS (*N* = 25) [19]. Relevant questions from these 3 interview guides are included in Supplementary Table 1.

### Concept elicitation interviews

Parents/caregivers of children with FXS (*N* = 10) participated in a set of structured, web-based daily journaling exercises for 5 days, allowing them time to consider the emotional experiences that shaped their decisions and actions, followed by in-depth 60-minute telephone interviews. Given the low prevalence of FXS, a nonprobabilistic approach was used to recruit the caregivers via a patient advocacy group for FXS that agreed to display an invitation on their website explaining the purpose of the study and providing contact details for the research team. Caregivers were eligible if they resided in the US, were the primary caregiver of a child (aged 3–18 years) with a confirmed diagnosis of FXS, had the primary role in making treatment decisions for a child, and were able to communicate proficiently in English. The study was double-blinded, in that the participants did not know the identity of the sponsor and vice versa.

The interviews employed a semi-structured guide comprising open-ended questions designed to elicit the caregiver’s experience of caring for a child with FXS. Follow-up probes were used as needed to ensure a full description of topics of interest (e.g., symptoms) was elicited. The guide was organized into 4 sections: (1) Background and presentation of diagnosis; (2) Professional healthcare management and symptomatology; (3) Emotional journey; and (4) Treatment experience. The contributions from the journaling exercises were used to tailor Sect. 3 (Emotional Journey) to each participant in order to further expand upon key concepts related to the individual caregiver’s experiences and perceptions while caring for a child with FXS.

Interviews were conducted by 2 experienced qualitative researchers with at least 10 years of experience in qualitative research focused on health care. Researchers were trained on the goals of the interview and topics of interest before completing their first interview. Prior to the start of each interview, all caregivers consented for the telephone interviews to be recorded for data collection purposes. Interviews were transcribed verbatim for qualitative analysis. Caregivers were compensated for completing the journaling exercises and the in-depth interview.

Qualitative coding and analysis for the in-depth interviews were consistent with best practices [20] and employed a hybrid deductive-inductive approach in line with principles of applied thematic (content) analysis [21]. A coding template outlining each code, and a description of its meaning was developed based on the semi-structured interview guide and journaling exercises; the template was updated as new concepts and/or themes emerged during the coding process. For the in-depth interviews, transcripts were independently coded by 2 coders in 3 rounds using an exhaustive approach such that saturation, the point at which no new concepts emerged, was achieved in the third round.

### Caregiver survey

At entry into CONNECT-FX, a total of 212 caregivers completed a Caregiver Survey that consisted of an open-ended question asking them to describe the 5 behavioral or emotional problems that had most impacted their child and family over the past year. The verbatim responses were recorded for qualitative analysis.

Qualitative coding and analysis for caregiver surveys were also consistent with best practices [20] and followed principles in line with applied thematic (content) analysis [21] and employed a deductive-inductive approach. The final coding template from the in-depth interviews, augmented with additional codes based on the ABC-C item descriptions in the ABC-C user manual, informed initial analytic domains. Responses to the open-ended caregiver survey questions were iteratively coded by 2 experienced qualitative researchers, and reconciliation meetings were held during the coding process to reconcile discrepancies and update the coding template as new concepts and/or themes emerged during the coding process.

### Cognitive interviews

Caregivers of children with FXS (*N* = 25) participated in a 90-minute semi-structured cognitive interview via video conference. Caregivers were recruited using a nonprobabilistic approach through a recruitment vendor supplemented by a patient advocacy group. Outreach methods included emails to caregivers of patients with FXS, as well as posts to various social media platforms and websites. Caregivers were eligible if they resided in the US, were the parent/primary caregiver of a child (aged 3–18 years) with genetically confirmed FXS, reported at least 4 of 7 specified problem behaviors included on the ABC-C (“aggressive to others”, “temper tantrums”, “doesn’t sit still”, “prefers solitary activities”, “stares into space”, “repetitive hand/body/head movements”, “difficult to get through to”), and were able to communicate proficiently in English. Once the interview was scheduled, a study packet was shipped to participants that included the following: 2 copies of the informed consent form, the ABC-C, a sociodemographic form, and a pre-paid envelope for return of the materials to the study team. Informed consent was obtained prior to the interview, and the caregiver signed the form and returned 1 copy along with other study materials to the study team. All study procedures and materials were reviewed and approved by the Advarra Institutional Review Board.

Cognitive interviews were conducted by 2 experienced doctoral-level researchers with mixed-method expertise. Researchers were trained on the interview objectives and interview guide, including completion of at least 1 pilot interview. At the start of the interview, the researcher held an informed consent discussion, obtained written consent, and audio recorded verbal consent. During the interview, caregivers completed the entire 58-item ABC-C to familiarize themselves with the content of the instrument. Subsequently, caregivers were asked open-ended questions about the readability of instructions, comprehension of response options, and adequacy of the recall period. To minimize burden, each caregiver was cognitively interviewed on 16 of the 56 items comprising the FXS-specific portion of the ABC-C. To ensure that all items included the ABC-C FXS scoring algorithm were adequately addressed, a randomization method was used to distribute the items across 5 different interview versions. Each item was evaluated by a minimum of 4 participants, which is consistent with previous literature, where a minimum of 3 to 5 participants evaluating each item is common [22–26]. Caregivers were modestly compensated upon study completion.

Each cognitive interview transcript was professionally transcribed for qualitative analysis. A coding template was created in Microsoft Excel to systematically track caregivers’ responses regarding their interpretation of the ABC-C instructions, response options, and items to facilitate evaluation of literal, evaluative, and inferential comprehension of the ABC-C. The 2 researchers independently coded the content of the first 4 transcripts and then results were compared to ensure coding consistency. Any discrepancies were identified and reconciled by modification and/or re-definition of the coding template. Each of the remaining transcripts was coded by 1 of the 2 researchers using the revised coding template. Questions with discrete responses (i.e., yes/no or numerical ratings) regarding readability and appropriateness of response options and recall period were also put into table format and paired with verbatim supportive quotes for each participant.

A formalized system of assessment was employed to evaluate 3 aspects of caregivers’ comprehension of the ABC-C instructions and items: literal, inferential, and evaluative [27]. Literal comprehension (i.e., whether the participant displayed any confusion or misunderstanding about the question as written without further discussion) was assessed during the interview by the interviewer. Inferential comprehension (i.e., whether the caregiver was able to explain what the item meant in their own words) was assessed during qualitative analysis. Evaluative comprehension (i.e., whether the caregiver was able to form an opinion and answer questions based on the information provided) was determined based on the caregiver’s ability to provide a response to an item. Each caregiver needed to demonstrate a minimum of 2 of the 3 comprehension components to show adequate understanding of the questions and instructions. Adequate comprehension of the ABC-C_FXS_ in the overall sample was determined if ≥ 80% of caregivers showed comprehension in each of the 3 aspects [28].

## Results

Characteristics of the children with FXS and their caregivers across the 3 samples are shown in Table 1.

**Table 1:** Participant Characteristics

	Concept Elicitation	Caregiver Survey	Cognitive Interviews
*N*	10	212	25
*Individual With FXS*			
Age, years, mean (range)	10.2 (3–17)	9.7 (3–17)	10.3 (3–17)
Males, n (%)	8 (80)	159 (75)	22 (88)
Race/Ethnicity			
White			20 (80)
Hispanic/Latino			2 (8)
African American			1 (4)
Other			2 (8)
*Caregiver of Individual With FXS*			
Age, years, mean (range)	38 (25–54)	42.4 (28–53)	
Females, n (%)	10 (100)	22 (88)	
Relationship to child			
Mother	10 (100)	19 (76)	
Father		3 (12)	
Other family		2 (8)	
Professional caregiver		1 (4)	
Education, college or above, n (%)	NR	NR	21 (84)

FXS, Fragile X syndrome; NR, not reported

### Concept elicitation interviews

In the concept elicitation interviews (*N* = 10), the most commonly reported behaviors associated with FXS were: seeks isolation from others (*n* = 7; 70%), lack of interaction (*n* = 6; 60%), and aggression toward others (*n* = 6; 60%). Half of all caregivers reported that their child’s condition was associated with a lack of attention (*n* = 5; 50%), irritability (*n* = 5; 50%), and temper tantrums (*n* = 5; 50%). Behaviors associated with stereotypy (*n* = 4; 40%), hyperactivity (*n* = 4; 40%) and inappropriate speech (*n* = 3; 30%) were reported by less than half of all caregivers. Saturation for behavioral problems related to FXS was achieved by the eighth interview (Table 2).

**Table 2:** Concept elicitation interviews saturation table for behavioral problems associated With FXS^a^

Code	Caregiver ID										Total *n* (%)
	Round 1			Round 2				Round 3			
	1	2	3	4	5	6	7	8	9	10	
**Social Avoidance**											
Seeks isolation from others	**X**	X		X		X		X	X	X	7 (70)
Prefers to be alone	**X**			X					X	X	4 (40)
Prefers solitary activities					**X**					X	2 (20)
Elopement			**X**					X			2 (20)
**Socially Unresponsive/Lethargic**											
Lack of interaction	**X**			X		X		X	X	X	6 (60)
Lack of attention					**X**	X	X	X		X	5 (50)
Lack of movement	**X**		X	X							3 (30)
Resists physical contact	**X**								X	X	3 (30)
Depressed mood			**X**								1 (10)
**Misbehavior/Irritability**											
Aggressive to others	**X**			X		X	X	X	X		6 (60)
Irritability	**X**			X			X	X		X	5 (50)
Temper tantrums/outbursts	**X**		X			X		X	X		5 (50)
Screams/yells/cries inappropriately	**X**		X					X	X		4 (40)
Harming of others			**X**	X			X		X		4 (40)
Eating problem	**X**	X				X					3 (30)
Stubbornness	**X**					X		X			3 (30)
Self-injurious				**X**					X		2 (20)
Emotionally reactive								X	X		2 (20)
Disruptive			**X**								1 (10)
Disobedient								**X**			1 (10)
**Restrictive and Repetitive Behavior**											
Compulsive behavior					**X**	X		X		X	4 (40)
Repetitive speech		**X**		X				X			3 (30)
Transition issues				**X**		X				X	3 (30)
Repetitive motor movement		**X**									1 (10)
**# of new concepts**	**11**	**2**	**3**	**2**	**3**	**0**	**0**	**1**	**0**	**0**	**22 (100)**
**% of new concepts** (*n* = 22 concepts)	**50.0**	**9.1**	**13.6**	**9.1**	**13.6**	**0.0**	**0.0**	**4.5**	**0.0**	**0.0**	
**Cumulative % of new concepts**	**50.0**	**59.1**	**72.7**	**81.8**	**95.4**	**95.4**	**95.4**	**100.0**	**100.0**	**100.0**	

FXS, Fragile X syndrome

**Bold** indicates the first appearance of a concept

^a^To assess saturation (the point at which no new relevant or important information is gained by recruiting additional participants), transcripts were coded in rounds (i.e., first 3 transcripts in round 1, the next 4 in round 2 and the final 3 in round 3). Bolded X indicates when the concept was first reported in the interviews. Non-bolded X indicates the occurrence of the concept within the any interviews, or thematic prevalence [20]. Half of the new concepts (*n* = 11) were reported in the first interview with no new concepts being reported after the eighth interview indicating that saturation was reached in the concept elicitation interview sample

Most caregivers (*n* = 9; 90%) reported at least 1 behavior representative of social avoidance (e.g., self-isolating, wanting to be alone), as well as a broad spectrum of irritable behaviors, ranging from verbal (e.g., “talking back”, “hollering”) to physical aggression (e.g., “pushing”, “hitting”). Typical reports of behaviors related to social avoidance were preference for isolation, or preference for the company of single family members rather than groups of people, including friends. Socially avoidant behaviors had a negative impact on important activities, such as travel, schooling, or visits to the doctor, and manifested as elopement (e.g., “wandering off”, “actively trying to escape”:

*He doesn’t like to be out*,* doesn’t want to socialize too much. [CG1.1]*

*He would stay up in his room. He didn’t want to come downstairs. He didn’t want to be around people. [CG1.4]*

*She will at all costs avoid any type of new social engagement. [CG1.10]*

In terms of social unresponsiveness, caregivers generally reported that their child did not interact and wanted no involvement with other children. Caregivers reported that even when interacting socially or emotionally, “understanding how to maintain interaction” was challenging, or that their child may respond negatively to affection:

*He didn’t want to go outside and swing anymore…He didn’t want to become active. [CG1.4]*

*I can remember going back to when he was an infant and he didn’t make eye contact. [CG1.8]*

*[Children her own age] try to play with her and she doesn’t want anything to do with them. [CG1.9]*

Caregivers commonly described verbal irritability as taking the form of inarticulate “yelling” or non-verbal “screams,” often at inappropriate times. This behavior, as well as others stemming from irritability, could make “control” difficult, both for the caregiver and the child, and were compounded by the child’s inability to communicate or “explain himself.” “Stubbornness,” an inability to “manage” themselves, and general uncooperative behavior impacted other activities, including participation in family activities, general schooling, or visits to the doctor:

*His speech is not great*,* so when he starts having a lot of the behaviors is when he’s trying to explain himself and we may not understand him; that’s when we start seeing more aggression. Or*,* him being upset or just being really angry or just stubborn. That’s one thing*,* he’s really stubborn. Again*,* it’s part of not being able to explain himself sometimes. [CG1.1]*

*We knew he needed help to calm down the brain*,* it would be so he wouldn’t have as much aggression towards anybody. [CG1.4]*

*Wrong emotion sometimes or the wrong…yes. He’ll be playing and tickling and everything’s great*,* and then*,* all of a sudden*,* he’s like*,* “I hate you*,*” and running away mad and angry. It’s like*,* “Where did that come from?” [CG1.8]*.

### Caregiver survey

The themes identified in the responses to the caregiver survey (*N* = 212) were generally consistent with the results from the concept elicitation interviews (Table 3). Most caregivers reported at least 1 behavior associated with irritability (*n* = 170; 80.2%). The most prevalent irritable behaviors included aggression toward others (e.g., “hitting”, “biting”) when they do not want to comply or are overwhelmed (*n* = 91; 42.9%), irritable/whiny behaviors in response to transitions and frustration (*n* = 61; 28.8%), and temper tantrums when their demands are not immediately met (*n* = 61; 28.8%):

**Table 3:** Caregiver survey: spontaneously elicited behavioral problems (*N* = 212)

Observed Behavioral Problem	*n* (%)
**Irritability**	**170 (80.2)**
Aggressive/aggression	91 (42.9)
Irritable/whiny	61 (28.8)
Temper tantrums/outbursts	61 (28.8)
Disobedient/uncooperative	42 (19.8)
Self-harming behavior	39 (18.4)
Screaming/crying inappropriately	31 (14.6)
Demands must be met immediately	28 (13.2)
Yells inappropriately	26 (12.3)
Mood changes quickly	19 (9.0)
Inappropriate behavior/lack of boundaries	13 (6.1)
Controlling behavior	8 (3.8)
**Anxiety**	**138 (65.1)**
**Hyperactivity**	**120 (56.6)**
Easily distracted	61 (28.8)
Excessive activity/restless/can’t sit still	59 (27.8)
Impulsive	29 (13.7)
Endangering behavior	20 (9.4)
Disturbs others	19 (9.0)
**Socially Unresponsive/Lethargic**	**107 (50.5)**
Difficulty making friends/lack of friends	38 (17.9)
Doesn’t pay attention to instructions	29 (13.7)
Doesn’t use words/gestures to communicate	17 (8.0)
Difficult to get through to	16 (7.5)
Few social reactions to others	14 (6.6)
Unresponsive to structured activities	10 (4.7)
Inactive/stays in one position	10 (4.7)
Unresponsive to social play	9 (4.2)
Depressed mood	7 (3.0)
Preoccupied/stares into space	4 (1.9)
Fixed expressions/lacks emotional responses	4 (1.9)
Resists physical contact/affection	3 (1.3)
**Social Avoidance**	**103 (48.6)**
Seeks isolation from others	49 (23.1)
Prefers to be alone/solitary activities	47 (22.2)
Elopement	33 (15.6)
**Stereotypy**	**64 (30.2)**
Odd behavior	42 (19.8)
Recurring/repetitive body movements	30 (14.2)
**Inappropriate Speech**	**59 (27.8)**
Repetitive speech/questions	55 (25.9)
Talks excessively	6 (2.8)
Talks loudly to self	4 (1.9)
**Other**	
Communication issues	49 (23.1)
Transition issues	37 (17.5)
Impacts on family	29 (13.7)
Obsessive behavior	25 (11.8)
Development delays	22 (10.4)
Hyperarousal	11 (5.2)

*Temper tantrums. [Name’s] wants have to be met immediately or he becomes very angry and loud. He punches his brother and bites him. [138 − 0109]*

*Sometimes [name] can become very upset and frustrated and [name] can be very sensitive and starts crying. [144 − 0119]*

Almost two-thirds of caregivers (*n* = 138; 65.1%) attributed problematic behaviors, such as irritability or social avoidance, to their child being anxious or experiencing anxiety in new or social situations:

*Anxiety that causes aggression. If everyday routine is interrupted in anyway*,* it results in anxiousness which leads to hitting*,* spitting*,* kicking*,* shouting. [139–1015]*

*If his routines are interrupted*,* or in new settings*,* we can see behaviors such as laying down on the floor…when he can’t adjust to a change*,* he is irritable and/or aggressive for much of the day—daily issue. [147 − 107]*

*Anxiety—hiding under his bed*,* behind chairs*,* or people to avoid a person or situation*,* usually while screaming. [136 − 0120]*

Over half of the caregivers (*n* = 120; 56.6%) reported that their child exhibited hyperactive behaviors, including being easily distracted (*n* = 61; 28.8%) and/or restless (*n* = 59; 27.8%), which often had a negative effect on daily functioning (e.g., completing schoolwork or chores):

*Problems staying on task or following directions at home with chores or doing schoolwork*,* forgetting items that are due*,* and having trouble keeping track of supplies. [145 − 0102]*

*Inability to focus/concentrate—does not sit still and attend to tasks. Makes it difficult at school and affects his learning. [150 − 0112]*

Approximately half of the caregivers reported at least 1 problematic behavior indicative of social unresponsiveness (*n* = 107; 50.5%) and/or social avoidance (*n* = 103; 48.6%). Socially avoidant behaviors included seeking isolation from others, preferring to be alone, and elopement:

*At family events*,* [name] often refuses to join in and often finds an activity away from the group. [129 − 107]*

*Escapes public places in avoidance. Escapes home when is denied a preferred activity. [139 − 103]*

Social unresponsiveness was generally described by caregivers as their child not wanting to interact with other people, including peers, and was attributed to a lack of “social skills.”

*He started school 10 months ago and has showed little interest in making friends. When other kids talk to him he looks like he wants to talk*,* but doesn’t have the language or social skills to do so. [150 − 0112]*

*Social skills/making and keeping friends. [Name] needs to learn to understand boundaries when engaging with peers. She has difficulty sharing and taking turns. [157 − 0102]*

Some caregivers reported problematic behaviors associated with stereotypy (*n* = 64; 30.2%) and inappropriate speech (*n* = 59; 27.8%). These behaviors were primarily related to repetitive movements and speech.

### Cognitive interviews

All caregivers (*n* = 25) demonstrated comprehension of the ABC-C instructions. The majority (*n* = 21; 84%) reported “no problems” with understanding the instructions as written:

*They want information from the caregiver*,* just wanting to know by you observing your child*,* how would you rate situations. [CG2.012]*

*[I]t’s basically consider the past week’s behavior and decide on a scale of zero to three*,* zero not a problem at all and then increasing in severity of problems. [CG2.025]*

The majority of caregivers (*n* = 21; 84%) could select a response without difficulty and reported that the items were easy to answer. Almost all caregivers (*n* = 22; 88%) reported that the response options made sense in terms of their child’s behavior.

*The wording is simple and it made sense that you have slight*,* moderate*,* and severe*,* you have different levels to pick or*,* of course*,* not at all—they were appropriate to me*,* easy to gauge. [CG2.002]*

*Yes*,* yes it does*,* it’s an easy way to—it’s a way to rate that I can kind of relate to it in my brain*,* from serious to this is not a problem for us at all. [CG2.007]*

The remaining caregivers (*n* = 3; 12%) reported that they understood the options provided but wanted more available response options.

Generally, caregivers reported that when determining which response option to select, they considered how frequent the behavior was, how their child’s behavior affected other family members, if it was disruptive to the child’s or family’s daily life, or if their child’s relationships were negatively affected.

*Does the behavior occur*,* yes or no*,* is it a problem that it occurs*,* yes or no*,* and then how severe is that problem… if it is disruptive to our family or other kids or property or himself. [CG2.009]*

*I think about the frequency and how the behavior impedes upon his normal everyday functioning. [CG2.024]*

When asked to rate the readability of the ABC-C_FXS_, the vast majority of caregivers (*n* = 22; 88%) indicated that the instrument was easy to read and answer:

*It was pretty easy to answer; so much applies to him that I understood the question*,* because they were things I was familiar with. [CG2.002]*

*[All] of it pretty much made sense. Yeah*,* whether my child has it or not*,* yeah*,* I understand them. [CG2.006]*

*I think the*,* what is that called a rubric or whatever*,* that you guys give is very descriptive and makes it clear what you’re asking*,* and that helps a lot. So it doesn’t leave a lot of guess work for me. [CG2.008]*

More than three-quarters of the caregivers (*n* = 19; 76%) found the 7-day recall period of the ABC-C_FXS_ appropriate for reporting on their child’s behavior:

*Yeah*,* I think so. I don’t know if I could go much further past that*,* but*,* yeah*,* a week is appropriate. [CG2.002]*

*I would say a week is a good timeframe to decide; I mean*,* any more maybe things will start running together. [CG2.005]*

*So I think seven days, the information is still fresh in your mind. [CG2.013]*

*I think you can get a good snapshot of your child in a seven-day period. [CG2.015]*

All caregivers showed item comprehension, i.e., demonstrating at least 2 of 3 aspects of comprehension for each item. Overall, ≥ 80% of caregivers showed comprehension for each item in all evaluated aspects of comprehension. Quotes that support comprehension are provided in Supplementary Table 2. All caregivers reported that they had no difficulty interpreting the response options:

*The wording is simple and it made sense that you have slight*,* moderate*,* and severe; you have different levels to pick or*,* of course*,* not at all—they were appropriate to me*,* easy to gauge. [Z002]*

*I like the response options in terms of zero*,* slight*,* moderate*,* or severe. [Z017]*

*Yes*,* yes*,* it does*,* it’s an easy way to—it’s a way to rate that I can kind of relate to it in my brain*,* from serious to this is not a problem for us at all. [Z007]*

## Discussion

This research aimed to establish the content validity of the ABC-C_FXS_ for the measurement of behavioral symptoms associated with FXS. The qualitative evidence supports the content validity of FXS-specific scoring algorithm with 6 subscales (Social Avoidance, Irritability, Socially Unresponsive, Stereotypic Behavior, Hyperactivity, and Inappropriate Speech) for measuring clinical trial endpoints for future potential treatments for FXS. Findings from the cognitive interviews showed that the ABC-C_FXS_ was well understood and easily interpreted by caregivers. Taken together, this qualitative evidence supports the use of the ABC-C_FXS_ to assess behavioral symptoms in children with FXS with the content of the Social Avoidance, Irritability, and Socially Unresponsive subscales being most relevant to caregivers of children with FXS.

The qualitative evidence from the concept elicitation interviews and caregiver survey showed that caregivers shared similar experiences regarding their observations of their child’s behaviors. The socially avoidant behaviors reported by caregivers generally corresponded to those items on the ABC-C_FXS_ Social Avoidance subscale. Similarly, aspects of misbehavior and irritability that were frequently reported overlap with items on the ABC-C_FXS_ Irritability subscale. Lack of interaction and lack of attention are represented by items on the ABC-C_FXS_ Socially Unresponsive/Lethargic subscale items. The most frequently reported behavioral symptoms, grouped into domains of Misbehavior/Irritability, Hyperactivity, Social Avoidance, Socially Unresponsive/Lethargic, Stereotypy, and Inappropriate Speech, generally correspond to the behavioral domains included in the ABC-C_FXS_. Additionally, the ABC-C_FXS_ was well understood, made sense with regard to the behavioral challenges caregivers experienced while caring for a child with FXS, and had interpretable response options. The instrument’s recall period of 1 week was considered appropriate by more than three-quarters of the caregivers.

The sociodemographic profile across the qualitative studies presented here is similar to previous reports based on the Fragile X Online Registry With Accessible Research Database in the United States [29, 30], the principal resource of clinical and sociodemographic information on individuals with FXS in the US. Additionally, all caregivers in the cognitive interview study had some level of post-secondary education, which coincides with previous reports involving caregivers of children with FXS [31–33]. Taken together, the sociodemographic profile of the population included in the present work is comparable to that of other research in FXS and consequently strengthens the generalizability of the current findings.

In conclusion, the ABC-C_FXS_ is fit-for-purpose for measuring clinical trial endpoints in FXS. The content validity evidence from both qualitative interview studies supports the use of the ABC-C_FXS_ to assess behavioral problems in children with FXS.

## Electronic Supplementary Material

Below is the link to the electronic supplementary material.

Supplementary Material 1

## Data Availability

All relevant data generated and/or analyzed during this study are included in this published article.

## Footnotes

1 Fit-for-purpose in the regulatory context is equivalent to *valid* within modern validity theory (i.e., “the degree to which evidence and theory support the interpretation of test scores for the proposed use”.

## Abbreviations

ABC-C: Aberrant Behavior Checklist-Community
ABC-C_FXS_: Fragile X syndrome-specific portion of the Aberrant Behavior Checklist-Community
CE: Concept elicitation
CS: Caregiver Survey
COA: Clinical outcome assessment
CONNECT-FX: Clinical Study Of caNNabidiol in childrEn and adolesCenTs With Fragile X
FDA: Food and Drug Administration
FXS: Fragile X syndrome
ObsRO: Observer-reported outcome
US: United States
