# Supplementary material for: Caregiver-perceived behavioral challenges in fragile X syndrome and implications for measuring treatment benefit in clinical trials
Source: J Patient Rep Outcomes. 2026 Apr 1;10:77. doi: 10.1186/s41687-026-01054-9 (PMC13168368; doi:10.1186/s41687-026-01054-9)
Supplement: Supplementary file 1 — Supplementary Material 1 [file 41687_2026_1054_MOESM1_ESM.docx]

**Supplementary Data**

**Supplementary Table 1. Relevant Questions From Interview Guides**

**Concept Elicitation Interview Questions**

**Section 1: Background (Presentation/Diagnosis)**

- 1. What symptoms did you first become aware of/did [child] first suffer from?
  2. What symptoms and/or events prompted you to consult with a physician?

**Section 2: HCP Management & Symptomatology**

1. Have [child’s] symptoms changed over time?
   1. [IF YES] Which symptoms have gone away or decreased over time? [PROBE: age, severity]
   2. [IF YES] Have any symptoms worsened over time? [PROBE: age, severity]
2. Which symptoms is [child] suffering from now? (PROBE: hyperactivity, attention-deficit, aggression (self/outward), depression, anxiety, socialization, etc.)
   1. Which of these symptoms bother [child] the most today?
      1. Why those?
      2. Is [child] receiving treatment for these symptoms? If so, what?
   2. Which of [his/her] symptoms are the most challenging for *you* to manage?
      1. Why those?

**Section 3: Emotional Journey**

1. Now we want to learn more about how FXS impacts your daily life.
   1. To begin, please take me through you and [child’s] daily routine? Take your time and please share as much detail as you can.
      1. How have you tweaked your routine to accommodate FXS?
   2. What are the most significant frustrations you face day-to-day? [PROBE: impacts to social life, occupational/job, economic, emotional, financial, family dynamics, etc.]
   3. What do you consider to be the biggest day-to-day challenges in caring for [child]? [PROBE: symptomatology, HCPs, resources, etc.]
   4. What do you wish you could tell everyone about being a [mother/father] of a child diagnosed with FXS that most people don’t know or can’t see?

**Caregiver Survey Question**

What are the five behavioral, emotional, or social problems that most impacted your son/daughter and his/her family in approximately the past year? Please provide as much detail as possible to convey the nature, severity, and frequency of the problems.

**Cognitive Interview Questions**

*Interviewer asks the participant to read and complete the ABC-C. Interviewer allows the participant to complete before proceeding.*

Now that you’ve completed the questionnaire, I’d like to ask you about your overall impressions of the questionnaire. After that, I would like you to walk me through some of the questions themselves so I can get a better idea of your thinking when you were answering these questions.

1. What do you think of this questionnaire?
2. On a scale of 0 to 10, with 0 being “very easy” and 10 being “very difficult,” please tell me how easy or difficult it was to answer these questions.
3. Is the wording of the questionnaire easy to understand?
4. Did you have difficulty reading any of the words?

Let’s look at the instructions.

1. In your own words, what are these instructions asking you to do?
2. Are the instructions clear?
3. Do you feel you have a good understanding of these instructions?
4. Was there anything confusing or difficult to interpret about the instructions?

Next, I’d like to look at the response options.

1. What do you think of the wording of the response options?
2. Do you think the response options are easy or hard to understand?
3. Do they make sense to you in terms of how you think about your child’s behavior?
4. What do you consider when deciding if a behavior is a problem?
5. Thinking in terms of how you would use these response options, what is the difference between a behavior that is a slight problem and a behavior that is a moderately serious problem?
6. What about the difference between a behavior that is a slight problem and a behavior that is not a problem?
7. What about the difference between a behavior that is a moderately serious problem and a behavior that is a severe problem?

Let’s look at the time period this questionnaire required you to reflect on**.**

1. Did you find this amount of time, 7 days, easy to remember?
2. Generally speaking, is this recall period appropriate for reporting on your child’s behavior?
3. Is there anything that could change how easy or hard it would be to give answers for the last 7 days?

Now I’d like to go through some of the individual questions in the questionnaire. For each question, I’m going to ask you to tell me what you think of it, to use your own words to explain what the question is asking, and also to walk me through your thinking when you answered it.

*Note to interviewer: For the Social Avoidance subscale, go through all 4 items with each participant. Additionally, 4 items from the Irritability subscale, 3 items from Socially Unresponsive/Lethargy, 2 items from Hyperactivity, 2 items from Stereotypy, and 1 item from Inappropriate Speech should be talked through with each participant. Items will be presented on a rolling basis from interview to interview. A written schedule of items to ask about in each interview will be developed based on interview appointment times and will accompany this interview guide.*

For each item, ask:

1. What did you think of this question?
2. What was it asking, in your own words?
3. How did you determine your response to this question? What did you consider?

**Supplementary Table 2. Results of the Cognitive Interviews of the ABC-C_FXS_**

| **ABC-C_FXS_ Domain** | **ABC-C_FXS_ Item** | | **Comprehension Supportive Quote** |
| --- | --- | --- | --- |
| Social Avoidance | 5 | Seeks isolation from others | “*I would explain it as anytime when he’ll segregate himself away from a situation.” [CG2.005]* |
|  | 16 | Withdrawn, prefers solitary activities | *“He definitely tends to do things on his own versus with people, so that was a moderate.” [CG2.016]* |
|  | 30 | Isolates himself/herself from other children and adults | *“Just wants to be alone, not wanting to be around other people, go off on his own.” [CG2.012]* |
|  | 42 | Prefers to be alone | *“It just means that the child prefers to be alone versus being in a crowd or being even in the room with someone.” [CG2.002]* |
| Hyperactivity | 1 | Excessively active at home, school, work or elsewhere | *“Just not sitting down, always up doing something, intruding and evading other people’s space, uncontrollable.” [CG2.012]* |
|  | 13 | Impulsive (acts without thinking) | *“Maybe he doesn’t mean to do it, it just happens.” [CG2.013]* |
|  | 15 | Restless, unable to sit still | *“[J]ust constantly moving, fidgeting, moving about, just can’t be in one place, motionless.” [CG2.025]* |
|  | 31 | Disrupts group activities | *“Can [they] work in a group together.” [CG2.016]* |
|  | 38 | Does not stay in seat (e.g., during lesson or training periods, meals, etc.) | *“Does he sit appropriately when he needs to?” [CG2.023]* |
|  | 39 | Will not sit still for any length of time | *“They won’t sit and watch television, they won’t sit and read a book, they are constantly up and down, up and down.” [CG2.012]* |
|  | 44 | Easily distractible | *“Easily distractible I said was a slight problem because all you have to do is have a doorbell go off or someone comes home or leaves or anything like that and he’s changed directions.” [CG2.009]* |
|  | 48 | Constantly runs or jumps around the room | *“[N]ot just was he having difficulty with attention, but is he actually running, jumping, and physically having difficulty with that attention piece.” [CG2.010]* |
|  | 54 | Tends to be excessively active | *“It's doing an action possibly repetitively or excessively, maybe excited about something.” [CG2.016]* |
| Irritability | 2 | Injures self on purpose | *“Intentionally harms oneself.” [CG2.001]* |
|  | 4 | Aggressive to others (verbal or physical) | *“Cursing at others, maybe lashing out like to hit or try to bite is what I envision when I'm thinking of verbal or physical aggression.” [CG2.017]* |
|  | 7 | Boisterous, inappropriate, noisy/rough | *“Wild, is your kid wild.” [CG02.014]* |
|  | 8 | Screams inappropriately | *“[D]oes he scream when that’s an inappropriate reaction to something.” [CG02.010]* |
|  | 10 | Temper tantrums/outbursts | *“Like how often does your child act out.” [CG02.001]* |
|  | 14 | Irritable and whiny | *“He's always, no matter what it is, if we're trying to get him to do something, is he resistant just with like I don't know… you know, like is he resisting in some sort of like minor way.” [CG02.018]* |
|  | 18 | Disobedient, difficult to control | *“I envision that it's doesn't follow instructions or doesn't behave appropriately in a given situation.” [CG02.017]* |
|  | 19 | Yells at inappropriate times | *“[I]nappropriate verbal behavior…” [CG02.024]* |
|  | 21 | Disturbs others | *“Not being able to keep to himself and is disruptive of others and bothersome.” [CG02.005]* |
|  | 24 | Uncooperative | *“I thought of how often does she follow directions the first time she’s asked, because I think if you have to ask really more than once then the child is not being cooperative.” [CG02.001]* |
|  | 29 | Demands must be met immediately | *“When he asks for something, do we need to do it right away or can we ask him to wait and see if he’ll wait patiently.” [CG02.023]* |
|  | 34 | Cries over minor annoyances and hurts | *“Cries over things that are just little things, like having to wait in line too long or something like that.” [CG02.002]* |
|  | 36 | Mood changes quickly | *“Did he go from happy to sad, angry to sad.” [CG02.014]* |
|  | 41 | Cries and screams inappropriately | *“I would describe it as does your child ever cry and scream at awkward times.” [CG02.005]* |
|  | 47 | Stamp feet or bang objects or slam doors | *“That’s a temper tantrum, stamps feet or bangs objects or slams doors, to me that’s aggression.” [CG02.011]* |
|  | 50 | Hurts self on purpose | *“Deliberately hurts himself or herself.” [CG02.013]* |
|  | 52 | Does physical violence to self | *“To me it’s the intent of physical violence to self, that’s how I interpret that one.” [CG02.010]* |
|  | 57 | Outbursts/tantrums when doesn’t get way | *“I was thinking more of looking for those full out meltdowns almost I guess you could say. When something didn’t go the right way, as opposed to maybe just hitting us twice and then being done with it, if he kept on going for five minutes or so.” [CG02.023]* |
| Socially Unresponsive/ Lethargy | 12 | Preoccupied, stares into space | *“Just sometimes [not] there, somewhere else, just thinking [maybe], but just not with the rest of the other kids or other people around him.” [CG02.012]* |
|  | 20 | Fixed expression, lacks emotional response | *“I would say lack of affect.” [CG02.024]* |
|  | 23 | Does nothing but sit and watch others | *“[D]oes he prefer to observe versus participate.” [CG02.010]* |
|  | 25 | Depressed mood | *“Basically, how often is she sad.” [CG02.001]* |
|  | 28 | Does not pay attention to instructions | *“[S]o he does not pay much attention to instruction, it has to be repetitive all the time, all the time, all the time.” [CG02.013]* |
|  | 32 | Sits/stands in one position for a long/time | *“Standing, yeah, just like a statue.” [CG02.007]* |
|  | 37 | Unresponsive to structured activities | *“I think even if worded ‘Does not participate in structured group activities’ would maybe be a little bit more layman’s terms.” [CG02.014]* |
|  | 40 | Is difficult to reach, contact/get through to | *“I would describe it as the ability of someone to verbalize with him and get his attention.” [CG02.005]* |
|  | 43 | Doesn’t communicate by words/gestures | *“I would say maybe unwilling to communicate by words or movement.” [CG02.Z003]* |
|  | 51 | Pays no attention when spoken to | *“[H]ow responsive he is when you're talking to him, especially when you feel like you should have his attention.” [CG02.018]* |
|  | 53 | Inactive, never moves spontaneously | *“Sits still for extended periods of time.” [CG02.017]* |
|  | 55 | Responds negatively to affection | *“[D]oes he withdraw from any sign of affection; does he shrink away from a hug, does he stiffen when I hug him or put my hand on his shoulder or something.” [CG02.014]* |
|  | 56 | Deliberately ignores directions | *“I’m interpreting that as I know that he knew I was talking to him, I know that he knows what I’m asking him to do and he’s deliberately choosing not to.” [CG02.Z010]* |
|  | 58 | Shows few social reactions to others | *“When other people are trying to engage him, how responsive is he.” [CG02.018]* |
| Stereotypy | 6 | Meaningless recurring body movements | *“Sitting there maybe tapping the leg over and over and over and over.” [CG02.004]* |
|  | 11 | Stereotyped behavior, abnormal repetitive | *“[S]tereotyped behavior like flapping, repetitive movements, just to me perseverative type behaviors.” [CG02.025]* |
|  | 17 | Odd, bizarre in behavior | *“How bizarre, out of the ordinary behavior, out of the ordinary way of thinking.” [CG02.021]* |
|  | 35 | Repetitive hand, body, or head movements | *“They move certain parts of their head or body and it just keeps happening.” [CG02.008]* |
|  | 45 | Waves/shakes the extremities repeatedly | *“He doesn’t want to go bed, that’s when he starts waving his arms the most and shaking and going like this, moving back and forth violently because he wants what he wants.” [CG02.015]* |
|  | 49 | Rocks body back and forth repeatedly | *“I understand the question, rocks body back and forth repeatedly, but my son doesn’t do that, so I answered not a problem.” [CG02.002]* |
| Inappropriate Speech | 9 | Talks excessively | *“Always have something to say, always want to talk, want to be the center of trashing and talking, never waits to his turn.” [CG02.012]* |
|  | 22 | Repetitive speech | *“Repetitive, that means says the same thing over and over.” [CG02.014]* |
|  | 33 | Talks to self loudly | *“He like self-talks or sort of like inappropriately chattering to himself.” [CG02.018]* |
|  | 46 | Repeats a word or phrase over and over | *“[D]oes she say the same thing over and over again in the same like couple of minutes.” [CG02.001]* |
